# FOOD DESERT RESIDENCE AND COGNITIVE TRAJECTORIES IN US OLDER POPULATION AGED 65 AND OLDER

**DOI:** 10.1093/geroni/igad104.0351

**Published:** 2023-12-21

**Authors:** Boeun Kim, Sarah L Szanton, Roland Thorpe, Paris Adkins-Jackson, Deidra Crews, Laura Samuel

**Affiliations:** Johns Hopkins University, Baltimore, Maryland, United States; Johns Hopkins University, Baltimore, Maryland, United States; Johns Hopkins University, Baltimore, Maryland, United States; Columbia University, New York City, New York, United States; Johns Hopkins University, Baltimore, Maryland, United States; Johns Hopkins University School of Nursing, Baltimore, Maryland, United States

## Abstract

A food desert is defined as a census tract with limited access to affordable and nutritious foods. Inequitable access to healthy food may contribute to disparities in cognitive health, but little is known about the role of food deserts in cognitive health. We examined association between living in food deserts and cognitive function in older adults 65 years and older. These analyses employed cohort design by linking the US Department of Agriculture 2010 Food Access Research Atlas data to 2011-2021 National Health and Aging Trends Study. Orientation, executive function, and memory were assessed annually. Food desert residence at baseline was determined if more than 500 or 33% residents within a census tract had low income and low access to healthy and affordable foods. Weighted mixed-effects models were fitted among 5270 community-dwelling older adults without dementia at baseline. Mean baseline age was 76.7 (±7.4) years and 10.7% participants (n = 659) lived in food deserts. Black and Hispanic people and individuals with lower educational attainment were more likely to live in food deserts. At the age of 80 years, individuals living in food deserts showed orientation and executive function scores that were -0.26 points (95% CI: -0.48, -0.03) and -0.13 points (95% CI: -024, -0.01) lower than their peers, respectively. Living in food deserts was also associated with 0.05 points faster annual decline (95% CI: -0.09, -0.01) in orientation. Residence in food deserts is associated with worse cognition in older age and could be a driver of disparities in cognitive health.

